# Recalcitrant sterile keratolysis of corneal graft associated with systemic flare-up of rheumatoid arthritis: A case report and review of literature

**DOI:** 10.22336/rjo.2026.02

**Published:** 2026

**Authors:** Manasi Tripathi, Shraddha Sneha, Manpreet Kaur, Ashish Markan

**Affiliations:** 1 Dr. Rajendra Prasad Centre for Ophthalmic Sciences, All India Institute of Medical Sciences, Delhi, India

**Keywords:** keratolysis, graft melt, rheumatoid arthritis, biologics, AC = Anterior chamber, ALP = Alkaline Phosphatase, ALT = Alanine Transaminase, AMT = Amniotic Membrane Transplant, ANCA = Anti-neutrophil cytoplasmic antibody, AST = Aspartate amino-transferase, BCL = Bandage Contact Lens, CDVA = Corrected Distance Visual Acuity, CRP = C-Reactive protein, ESR = Erythrocyte sedimentation rate, DMARDs = Disease-modifying antirheumatic drugs, HCQ = Hydroxychloroquine, IOP = Intraocular pressure, KOH = Potassium hydroxide, LE = left eye, NSAID = Non-steroidal anti-inflammatory drugs, PCR = Polymerase Chain Reaction, POD = Postoperative day, PUK = Peripheral Ulcerative Keratitis, RA = Rheumatoid Arthritis, RE = right eye, TNFα = Tumor necrosis factor alpha, UDVA = Uncorrected distance visual acuity

## Abstract

**Purpose:**

To report a rare case of recalcitrant sterile corneal graft melt following routine optical penetrating keratoplasty in a patient with rheumatoid arthritis (RA), and to highlight the role of systemic disease exacerbation and tumor necrosis factor-alpha (TNF-α) inhibition in stabilizing the condition.

**Methods:**

A 54-year-old female with well-controlled RA underwent an optical triple procedure for a visually significant central adherent leukoma. Postoperative follow-up revealed recurrent sterile keratolysis of the donor cornea coinciding with systemic flare-ups of RA. A standard microbiological and serological workup was performed to rule out infectious and other systemic causes.

**Results:**

Despite appropriate topical and systemic therapy, including corticosteroids and re-suturing with amniotic membrane transplant, the corneal melt persisted and recurred. All infectious causes were ruled out. A significant rise in inflammatory markers correlated with systemic RA activity. Initiation of adalimumab led to stabilization of the corneal melt within two weeks, along with marked improvement in systemic symptoms. At 6 months, the graft remained opaque but tectonically stable, and the globe’s structural integrity was preserved. No recurrence was noted at 18-month follow-up.

**Discussion:**

Sterile corneal melt has been described in association with surgical interventions, drug toxicity, and systemic autoimmune diseases, particularly rheumatoid arthritis (RA), where dysregulated inflammatory pathways lead to keratolysis, typically presenting as peripheral ulcerative keratitis. In contrast, our case is unique in that keratolysis was confined predominantly to the donor corneal graft and graft–host junction following an otherwise uneventful optical penetrating keratoplasty. Infectious causes, NSAID toxicity, dry eye disease, and Sjögren syndrome were excluded. At the same time, the selective involvement of the donor tissue, negative microbiology, and elevated rheumatoid factor and inflammatory markers supported an immune-mediated graft melt related to rheumatic eye disease. Unlike in previously reported cases after lamellar or endothelial keratoplasty, in which melts originated in the host cornea or were associated with epithelial healing defects or Sjögren syndrome, our patient had complete initial graft epithelialization and normal ocular surface parameters. The temporal association of graft melt with a systemic RA flare, high RA factor titers, and involvement of the graft–host junction suggests a strong link between systemic inflammatory activity and graft survival. This case highlights a rare presentation of autoimmune-mediated sterile graft melt after routine penetrating keratoplasty. It underscores the importance of recognizing systemic disease activity as a critical factor in postoperative corneal outcomes.

**Conclusion:**

This case highlights the potential for sterile graft melt to manifest as a systemic autoimmune flare in RA. Early recognition and multidisciplinary management, including biologic agents such as adalimumab, may be crucial for preserving ocular integrity in such recalcitrant cases.

## Introduction

Ophthalmic manifestations of rheumatoid arthritis (RA) range from benign conditions, such as dry eye disease and episcleritis, to vision- and globe-threatening complications, including peripheral ulcerative keratitis (PUK) and scleromalacia perforans [1]. Sterile corneal melt is a rare but documented complication of rheumatic diseases.

Occasionally, topical therapy, disease-modifying antirheumatic drugs (DMARDs), and systemic steroids may be insufficient to manage the ocular morbidity resulting from these disorders, therefore necessitating the use of biologic agents. We hereby report a case of spontaneous recalcitrant sterile melt of a corneal graft associated with systemic flare-up of rheumatoid arthritis and its management.

## Case report

A 54-year-old lady, with RA well controlled on oral Hydroxychloroquine (HCQ), presented with complaints of diminution of vision in the right eye (RE) over the last 1 year. There was a history of bacterial keratitis in her right eye, which was medically managed in her hometown. Baseline examination and blood investigations are summarised in **Tables 1** and **2**, respectively. Following a complete preoperative work-up and consultation with her rheumatologist, the patient underwent the RE Optical Triple procedure.

**Table 1:** Preoperative ocular examination of the patient

	Right Eye	Left Eye
Lids and adnexa	Within normal limits	Within normal limits
Uncorrected distance visual acuity (UDVA)	Hand motions close to the face with accurate projection of rays	6/6
Intraocular pressure (IOP)	16 mmHg	18 mmHg
Slit lamp examination	4 x 5.5 mm central adherent leukoma involving the visual axis with cataractous lens	Within normal limits
Fundus	Media hazy, details not visible	Within normal limits
B Scan	Grossly anechoic vitreous cavity, no optic nerve head cupping	-
Schirmer’s test	10 mm	15 mm
Tear film break-up time	8 seconds	12 seconds
Corneal sensations	Normal and comparable in both eyes	

**Table 2:** Summary of blood investigations in the preoperative period, first episode of graft melt, and second episode of graft melt

	Preoperative baseline values	First episode of graft melt (2 weeks postoperatively)	Second episode of graft melt (4 weeks postoperatively)	Reference values
Hemoglobin (g/dL)	9.6	9.8	10.1	12-15
Hematocrit (%)	31.9	30.30	32.7	40-50
Red blood cell count (10^6/µL)	3.26	2.95	3.04	3.8-4.8
White blood cell count (10^3/µL)	6.11	11	9.79	4-10
Platelet count (10^3/µL)	196	293	268	150-410
Neutrophils (%)	68.4%	70.2%	**94.6%**	40-80
Lymphocytes (%)	23.4%	21.9%	4.4%	20-40
Eosinophils (%)	1.1%	0.1%	0%	1-6
Monocytes (%)	6.9%	7.6%	0.9%	2-10
Basophils (%)	4.18%	0.2%	0.1%	<1
Urea (mg/dL)	35	29.6	23	15-42
Creatinine (mg/dL)	1.8	1.8	1.6	0.52-1.04
Calcium (mg/dL)	8.7	7.8	8	8.4-10.2
Sodium (mmol/L)	137	142	146	137-145
Potassium (mmol/L)	3.8	3.3	3.8	3.5-5
Chloride (mg/dL)	108	117	120	98-107
Bilirubin (total)	0.25	0.4	0.45	0-1.2
ALT (U/L)	9	14	11	10-49
AST (U/L)	8	17	12	14-36
ALP (U/L)	108	111	106	46-122
Albumin (gm/dL)	3.5	3.8	3	3.5-5
Total protein (gm/dL)	7.4	7.3	3.9	6.3-8.2
Rheumatoid factor (IU/mL)	Not done	**256**	**325.3**	<14
ANCA	Not done	Not done	Negative	
CRP	Not done	2.4	**9.12**	1-5
ESR	20	**35**	**70**	<20

ALP = Alkaline Phosphatase, ALT = Alanine Transaminase, ANCA = Anti-neutrophil cytoplasmic antibody, AST = Aspartate amino-transferase, CRP = C-Reactive protein, ESR = Erythrocyte sedimentation rate

The early postoperative period was uneventful, and the patient achieved a UDVA of 6/24 at 1-week follow-up. Complete epithelisation of the donor graft was observed, and the ocular surface was within normal limits.

**Fig. 1:**
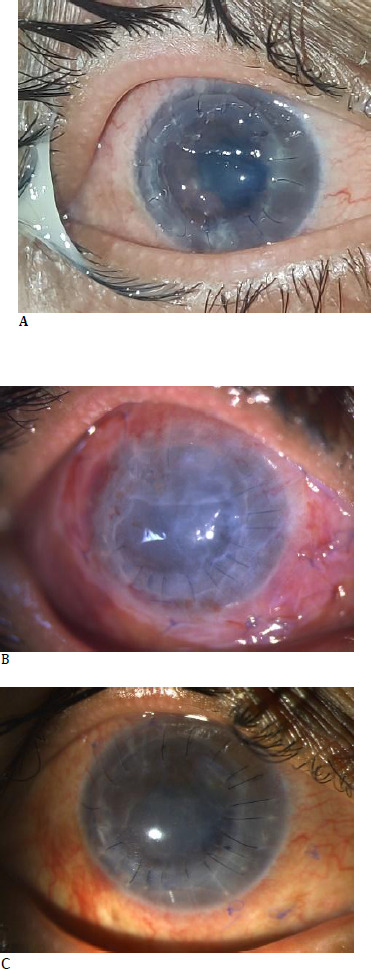
First episode of graft melt at 2 weeks postoperative period (**A**). A large central epithelial defect in the donor graft, with loose sutures and sterile infiltrates at the graft-host junction, should be noted. Conjunctival hyperemia, blepharitis, and mucoid discharge can also be observed. No corneal vascularization is observed. An amniotic membrane with an overlying bandage contact lens can be observed in the immediate postoperative period (**B**). On POD-10, marked improvement in the ocular surface is observed (**C**). The healed epithelial defect, resolution of conjunctival hyperemia, and improvement of blepharitis can be observed

However, at 2 weeks postoperative period, she presented to our emergency department with complaints of pain and diminution of vision in the right eye. The patient was severely photophobic with UDVA in RE 2/60 and IOP 10 mmHg. On examination, a large central epithelial defect was observed in the donor graft, with loose sutures and sterile infiltrates at the graft-host junction (**Fig. 1A**). Host cornea appeared stable and free from any epithelial defect or thinning. Siedle’s test was negative for wound leak at the graft-host junction. Blood investigations revealed increased rheumatoid factor (256 IU/mL) and elevated erythrocyte sedimentation rate (ESR: 35 mm/hr). She was urgently taken up for RE re-suturing of graft with amniotic membrane transplant (AMT) with bandage contact lens (BCL) placement. Postoperatively, topical Chloramphenicol 0.5% TDS, Prednisolone phosphate 1% 6 times a day, Cyclosporine 0.09% BD, topical sodium hyaluronate 0.15% 6 times a day, topical Hydroxypropyl methylcellulose (HPMC) 1% gel at night time, oral doxycycline 100 mg BD, oral vitamin C 500 mg QID and oral prednisolone (stepped up to 1.5 mg/kg body weight) were prescribed. There was symptomatic relief after the intervention, and stabilisation of the ocular surface was observed (**Fig. 1 B, C**).

**Fig. 2:**
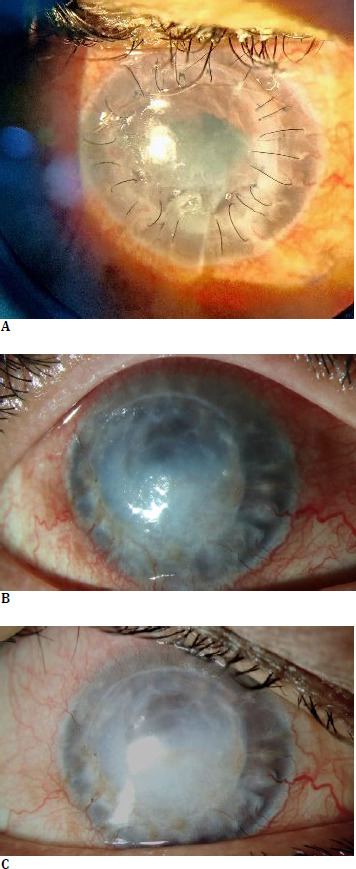
Second episode of corneal graft melt (**A**). The corneal infiltrates in the donor graft and host cornea near the graft-host junction should be noted. Infiltrates are denser in the donor graft than in the host. Corneal neovascularization is present at the graft-host junction. All sutures have loosened. In the inferotemporal quadrant, 8-0 vicryl suture applied during amniotic membrane transplant can be observed while the bandage contact lens (BCL) is inferiorly displaced. The clinical picture after two doses of Adalimumab (**B**). Corneal clarity has been lost after the second episode of graft melt. The corneal neovascularization, dilated tortuous conjunctival vessels, and blepharitis should be observed. The graft-host junction is intact, and the globe’s structural integrity is maintained. At 6 months postoperative follow-up, conjunctival hyperaemia has resolved, and the ocular surface appears stable (**C**). An adequate tear meniscus height is observed. Lid margins appear normal

Three weeks after the second intervention, she again presented with a repeat episode of graft melt. UDVA in RE was 0.5 m with finger counting, at an IOP of 6 mmHg. Anterior segment examination revealed loose sutures, infiltrates on the donor graft more concentrated near the graft-host junction, conjunctival congestion, and an inferiorly displaced BCL (**Fig. 2 A**).

AC shallowing was observed with a positive Seidel test at the inferior graft-host junction. This episode of ocular manifestations co-existed with acute exacerbation of systemic symptoms of rheumatoid arthritis – i.e., arthralgia, morning stiffness, and low-grade fever. Relevant ocular and systemic investigations were performed to ascertain the cause of this recalcitrant corneal melt. The conjunctival swab did not reveal any bacterial growth. Corneal scraping was performed, and the tissue obtained was subjected to microbiological assessment. No organism was isolated on Gram stain, 10% potassium hydroxide (KOH) mount, and bacterial and fungal culture. A tear sample was taken from the affected eye and subjected to polymerase chain reaction (PCR) to detect Herpesviridae DNA. This viral PCR was also negative. However, blood investigations revealed neutrophilia (94.6%) and markedly elevated rheumatoid factor (325.3 IU/mL). C-reactive protein (CRP: 9.12 mg/dL) and erythrocyte sedimentation rate (ESR: 70 mm/hr) were also elevated. A slight elevation of blood urea (2.2 mg/dL) was noted; however, other renal function tests and electrolyte profile were within normal limits. Serology was negative for hepatitis-B (HBsAg), human immunodeficiency virus (HIV-I and II), anti-HCV antibody, and syphilis. Chest X-ray revealed a normal radiological study.

The patient was urgently taken up for re-suturing of the corneal graft, anterior chamber re-formation, and repeat AMT with BCL placement. Oral Prednisolone was increased to 2 mg/kg to control both ocular and systemic exacerbations. However, the corneal graft melt remained recalcitrant, and there was no significant improvement in arthralgia. After consultation with her rheumatologist, Adalimumab (40 mg subcutaneous injection every 2 weeks) was added to the treatment regimen.

The corneal graft melt stabilized within 2 weeks of administration of the first dose of Adalimumab. Oral steroids were slowly tapered, and Adalimumab injection was continued for 12 weeks. Topical cyclosporine and lubricants, along with oral HCQ, were continued for maintenance. There was a marked improvement in ocular and systemic symptoms as well. However, graft clarity worsened over time (**Fig. 2 B**). Corneal sutures were removed as and when they appeared to have loosened. All sutures were out by 10 weeks after the second surgical intervention. As the corneal donor was successfully preserved through medical management, histopathological examination of the affected tissue was not feasible.

At 6 months postoperatively, CDVA RE was finger counting at close range, and IOP RE was 18 mmHg. Although the graft had opacified, the tectonic integrity of the globe was maintained (**Fig. 2 C**). Since the patient was comfortable and free of any ocular or systemic complaints, she refused further surgical intervention for visual rehabilitation. Routine follow-up evaluations were continued with both her treating ophthalmologist and rheumatologist, ensuring ongoing monitoring of her condition and coordination of care between specialties. There was no recurrence until 18 months postoperatively.

## Discussion

Sterile corneal melt has been reported with topical and oral NSAID abuse, after amniotic membrane transplant, implantation of intracorneal ring segments, trans-scleral diode laser, collagen cross-linking, and as a manifestation of systemic rheumatological disorders [2-5]. Autoimmune keratolysis secondary to rheumatic disorders, especially rheumatoid arthritis, is a rare but recognized complication [6]. The pathophysiology of corneal melt in RA is unclear but is thought to involve a hostile ocular surface and dysregulated inflammatory cytokines, leading to spontaneous keratolysis. These cases usually manifest as peripheral ulcerative keratitis because the peripheral cornea is close to conjunctival blood vessels and lymphatics, which subsequently deliver inflammatory cells and cytokines, perpetuating a vicious cycle of inflammatory corneal melt [6]. However, to the best of our knowledge and based on a thorough literature review, our case is unique in its presentation, as keratolysis was confined primarily to the donor corneal graft and the graft-host junction.

In our patient, Schirmer’s test was within normal values, and salivary secretions were satisfactory, making secondary Sjogren syndrome as the cause of graft melt unlikely. Corneal infections were ruled out. Topical non-steroidal anti-inflammatory drugs (NSAIDs) were not a part of the patient’s postoperative medications, thus ruling out NSAID-induced corneal melt as the cause. Topical fluoroquinolones are known to worsen corneal melts and perforations, but stopping moxifloxacin and switching to chloramphenicol did not stabilize the melt. The primary involvement of the donor graft, up to the graft-host junction, pointed towards a possible immune-mediated reaction. A negative infection profile, elevated rheumatoid factor, CRP, and ESR, supported the diagnosis of recalcitrant autoimmune graft melt as a manifestation of rheumatic eye disease.

Spontaneous sterile graft melt is a known complication in Boston type-1 keratoprosthesis; however, it is an undocumented occurrence following a routine, uneventful optical penetrating keratoplasty [7]. Al-Swailem et al. reported a case of acute sterile keratolysis after deep anterior lamellar keratoplasty (DALK) for keratoconus in a healthy patient who received a gamma-irradiated sterile cornea (GISC). Their case was managed by exchanging the GISC with a standard corneal tissue [8]. In contrast to their report, sterile keratolysis occurred within the standard donor cornea in our patient. Shan et al. reported two cases of sterile corneal melt after Descemet stripping endothelial keratoplasty [9]. However, they noted that the melt began in the host cornea rather than in the donor. They also noted delayed postoperative epithelial healing preceding the corneal melt. Additionally, both their patients were subsequently diagnosed with Sjogren Syndrome, which is a known cause of sterile corneal melt. In contrast to their report, our patient recovered well in the initial postoperative period after her optical triple procedure with complete epithelization of the graft. Moreover, her preoperative ocular surface work-up showed normal Schirmer’s test and TBUT, making dry eye or pre-existing Sjogren syndrome an unlikely precipitating factor. Shan et al. also report isolating Propionibacterium acnes and Corynebacterium pseudodiptheriticum in their cases. Still, they did not attribute the melt to these organisms because the microbiological findings did not correlate with the clinical picture and course of the disease. Williams et al. reported a case that mimicked sterile rheumatic corneal melt, in which typical signs of underlying infection were masked by rituximab-induced immunosuppression [10]. However, in our case, all ocular and systemic microbiological investigations were negative for any infectious organism.

In another case report, Newman et al. hypothesized that corneal graft rejection may be related to immune-mediated keratolysis and subsequent corneal graft melt [11]. The involvement of the graft-host junction and the occasional presence of AC cells in our case also suggested an immune-mediated reaction. However, histopathological confirmation could not be obtained because the graft tissue was successfully preserved. Numerous studies have demonstrated that polymorphonuclear neutrophils predominantly release proteolytic enzymes, reactive oxygen species, and matrix metalloproteinases, triggering inflammatory processes that may ultimately lead to corneal graft melting [12].

Another interesting finding was the simultaneous presentation of graft melt and a systemic flare-up of rheumatoid arthritis. A significant increase in blood inflammatory markers accompanied the exacerbation of systemic symptoms. Both scleritis and PUK associated with RA are known to frequently occur in patients with long-standing systemic disease and high titers of RA factor [13]. Our patient with sterile graft melts also had a long-standing disease (15 years) with high titers of RA Factor (325.3 IU/mL). These findings suggest a correlation between systemic activity and corneal graft survival after keratoplasty.

The lack of corneal vascularization at the onset of the disease makes us wonder whether inflammatory cells and mediators in the tear film or aqueous humour were responsible for the trigger. Of the two routes, tear film cells and mediators appear to be the likely culprits in fueling this inflammatory cascade, since the first episode of graft melt predominantly involved the anterior corneal layers, followed by total corneal involvement from the second episode onward. In retrospect, we believe that tear film cytokine profiling could have further substantiated the role of inflammatory mediators, such as TNF-α, in the pathogenesis. Tumor necrosis factor alpha (TNFα), a key proinflammatory cytokine, mediates apoptosis by increasing vascular permeability, promoting leukocyte migration, and enhancing adhesion molecule expression. In human corneal epithelial cell cultures, TNFα stimulates Matrix Metalloproteinase-9 activity, suggesting its role in the keratolytic pathway and its potential as a therapeutic target for corneal melts [14]. Adalimumab (Humira®, AbbVie Inc., North Chicago, IL, USA) is a fully human monoclonal antibody against TNFα. TNFα inhibitors, including adalimumab, are considered the first-line biologic therapy for RA [15]. Additionally, adalimumab has shown efficacy in managing peripheral ulcerative keratitis, making it a logical choice to address both systemic and ocular manifestations in our case. However, regardless of its beneficial effects, adalimumab carries a risk of serious adverse effects, such as increased risk of infections and malignancies, hepatotoxicity, leucopenia, and demyelinating disorders [16]. Therefore, a multidisciplinary approach, combined with continuous monitoring and follow-up, is crucial to optimize patient outcomes and ensure careful, judicious use of this biologic therapy.

### Learning points

- Sterile graft melt is a rare but devastating complication of keratoplasty.
- Active inflammation in rheumatoid arthritis can compromise the ocular surface.
- Systemic stabilization is essential to ensure a stable ocular surface, thus prolonging the survival of the corneal graft.
- Biologics may be useful for recalcitrant corneal graft melt associated with systemically active rheumatoid arthritis. A multidisciplinary approach is essential to manage these patients.
